# Food insecurity and its determinants in pastoralist and agrarian communities in South Omo Zone, Southern Ethiopia: a community-based cross-sectional study

**DOI:** 10.3389/fpubh.2024.1482208

**Published:** 2024-10-11

**Authors:** Mintesinot Melka Gujo, Lebitsi Maud Modiba

**Affiliations:** ^1^South Ethiopia Region Health Bureau Public Health Institute, Jinka, Ethiopia; ^2^Department of Health Studies, College of Human Sciences, University of South Africa, Pretoria, South Africa

**Keywords:** food insecurity, determinants, agrarian, pastoralist, Ethiopia

## Abstract

**Background:**

Despite the implementation of different interventions, food insecurity remains a major public health issue in rural areas of Ethiopia. However, there has been limited evidence regarding food insecurity and responsible factors in rural areas of Ethiopia, particularly in South Omo, Ethiopia. Hence, this study aimed to assess food insecurity and determinants in agrarian and pastoralist communities of South Omo Zone, Southern Ethiopia.

**Methods:**

A cross-sectional study was done among 605 randomly selected households in Benatsemay and South Ari districts from February 1 to 28, 2023. A standardized and validated Household Food Insecurity Access Scale (HFIAS) was used to measure food insecurity status. Data were entered using Epi-Info 7.1 and then transferred to SPSS V25 for analysis. To identify associated factors, a binary logistic regression model was employed. The strength of association was evaluated considering the adjusted odds ratio (AOR) and a 95% confidence interval (CI). A statistical significance was stated at *p*-value <0.05.

**Result:**

A total of 597 participants were involved in the study with a response rate of 98.7%. The overall prevalence of food insecurity using HFIAS was 42.2% (95%CI: 38.2, 46.3%), among which mild, moderate, and severe food insecurity accounted for 17.4, 16.6, and 8.2%, respectively. Of pastoralists, 114 (47.1%) were food insecure whereas 138 (38.9%) were food insecure in the agrarian. Food insecurity was affected by household head sex (AOR = 1.73, 95%CI: 1.14, 2.62), high dependency ratio (AOR = 2.53, 95%CI: 1.53, 4.20), no formal maternal education (AOR = 2.11, 95%CI: 1.07, 4.18), productive safety net program (AOR = 2.00, 95%CI: 1.16, 3.46) and land ownership (AOR = 1.80, 95% CI: 1.19, 2.72).

**Conclusion:**

Food insecurity was a significant problem in the study areas. Thus, it is crucial to improve female education, advance agricultural technologies, advocate family planning, and broaden productive safety net programs.

## Introduction

Food security is a state in which all people have consistent physical, social as well as economic access to safe, nutritious, and sufficient food that fits their dietary requirements and food choices to live a healthy and active life (1, 2). The fundamental elements of food security include the presence of food items, the financial ability to obtain food, the ease of obtaining food, and the consumption of enough food, which relies on the body’s ability to utilize nutrients effectively, alongside adequate dietary quality and safety of the food consumed (3, 4).

Household food security is confirmed when a household does not experience either chronic, which refers to the inability to consistently meet the minimum food requirements for a healthy life over three or more months, or transitory, which involves the inability to meet these requirements for less than 3 months (5, 6).

Food insecurity continues to be a serious public health issue worldwide, particularly in nations with lower incomes. Progress toward achieving everyone’s access to adequate food has also halted. The prevalence of moderate or severe food insecurity [sustainable development goals (SDG) Indicator 2.1.2] has remained substantially higher than pre-pandemic levels globally, with little improvement in 4 years following the rapid spike from 2019 to 2020 during the pandemic. In 2023, 2.33 billion people (nearly 30% estimated global population) were moderately or severely food insecure (7). Of this, 281.6 million population faced high levels of acute food insecurity (8). From 2020 to 2023, global food insecurity remained stable, but over 65 million people experienced moderate or severe food insecurity due to population growth. Globally, severe food insecurity increased from 9.1% in 2019 to 10.6% in 2020 and has been stable since then. In 2023, 10.7% of the world’s population (over 864 million people), were severely food insecure, putting their health and well-being at risk. Moderate or severe food insecurity was observed in 31.9% of rural areas, compared to 29.9% in peri-urban and 25.5% in urban areas globally (7).

Moderate or severe levels of food insecurity in Africa remained largely unchanged between 2022 and 2023. When compared to other regions of the world in 2023, Africa continues to have the highest proportion of its population enduring food insecurity. In 2023, 58.0% of Africa’s population was moderately or severely food insecure, about twice the global average, with 21.6% experiencing severe food insecurity. In 2023, 64.5% (313 million people) of Eastern Africa’s population was experiencing moderate or severe food insecurity, with 24.2% experiencing severe food insecurity (7).

Globally, Ethiopia faces severe food crises due to ongoing droughts, macroeconomic challenges, and internal conflict, with extreme weather driving livestock deaths and affecting household food and nutrition security, mainly in the pastoral areas of Southern Ethiopia. Between 2021 and 2022, acute food insecurity escalated in Ethiopia, affecting approximately 19.7 million people in 2023 (8). In developing nations like Ethiopia, rural communities face various problems in achieving food security, including multidimensional fluctuations in rainfall and temperature. During 2015/16, many Ethiopians were vulnerable to drought because of food insecurity, but the problem remains persistent in the country, particularly in the pastoralist setting (9, 10).

The 2023 Global Hunger Index (GHI) shows that since 2015 little progress has been made in reducing hunger. The 2023 global GHI score is 18.3, considered moderate (11). The world is still a long way from achieving SDG-2, Zero Hunger. After rising sharply from 2019 to 2021, global hunger has remained nearly constant for three consecutive years, affecting 9.1% of the population (735 million people) in 2023 compared with 7.5% in 2019. Africa has the highest proportion of the people facing hunger. In Africa, about 300 million people (20.4%) may have experienced hunger in 2023, and the number is still rising. By 2030, Africa will account for 53% of the world population suffering from hunger. Eastern Africa is home to over half (138.5 million) of Africa’s hungry people in 2023 (7). According to the 2023 GHI, Ethiopia is facing serious hunger (GHI score of 26.2) (11).

In terms of economic access to nutritious foods, more than one-third of the world’s population, or around 2.8 billion people, cannot afford a healthy diet by 2022. Inequalities are clear, with low-income countries having the highest proportion of the population unable to afford a healthy diet. The lack of advancement in food security and unsteady progress in economic access to healthy food put doubt on the possibility of attaining Zero Hunger globally, 6 years before the 2030 deadline. Moreover, the current lack of clear financing for food security and nutrition is hindering the achievement of SDG Targets 2.1 and 2.2 to end hunger, food insecurity, and malnutrition, but it needs higher and more cost-effective financing to meet the targets (7, 8).

There are regional variations in the extent of food insecurity in Ethiopia. According to a study done in the east Gojjam zone, northern part of Ethiopia, the magnitude of food insecurity was 10.71% (1). On the other hand, a study done in Sodo Town, the magnitude of food insecurity among households was 37.6% (10.8% mildly, 23.2% moderately, and 3.6% severely food insecure) (12). In addition to this, a study done in Borena, Ethiopia showed that the extent of food insecurity was 98.89% (13).

Food insecurity remains a significant public health issue in developing nations such as Ethiopia, especially in pastoralist settings. Ethiopia has been facing challenges in progressing either towards SDG Target 2.1, or Target 2.2 to end hunger, food insecurity, and all forms of malnutrition by 2030. Furthermore, most prior research was conducted in agrarian communities, whereas there is little evidence on determining factors for food insecurity and its determinants in pastoralist settings such as South Omo. As a result, food insecurity arises as a major public health issue in Ethiopia’s rural areas, particularly among pastoralist populations. However, understanding food insecurity and its determinants is highly demanding for evidence-based intervention and attaining SDG targets. Thus, the current study aimed to examine food insecurity and determinants among pastoralist and agrarian communities in Benatsemay and South Ari districts, Ethiopia.

## Materials and methods

### Study design, period, and setting

A community-based cross-sectional study was employed in both pastoralist and agrarian communities of South Omo Zone, Ethiopia. South Omo zone is found around 500 kilometers (KM) from Hawassa, the capital of the Southern Region, and 750 kilometers from Addis Ababa, the capital of Ethiopia (14). The South Omo Zone comprises 21 urban areas, 214 rural kebeles (the smallest administrative unit), 10 woredas (districts), and one city administration (Jinka town). Of these districts, six are pastoralist (Maale, Hammer, Salamago, Dasenech, Benatsemay, and Gnangatom), while the remaining four are agrarian (South Ari, Woba Ari, North Ari, and Bakadawula woredas).

According to the 2019 estimate of Ethiopia’s Central Statistical Agency (CSA), the population of the Zone was 802,467 (401,394 men and 401,073 women). There were an estimated 178,326 households in the South Omo zone. The usual yearly rainfall in the zone spans from 400 to 1,600 mm (14, 15).

Agriculture is the principal economic activity in the zone and the primary means of livelihood, with most people engaged in subsistence farming for personal use. The key crops cultivated in the study area are maize, sorghum, teff, coffee, vegetables, root crops, pulses, and oilseeds. The communities in the zone are mainly agro-pastoralists, raising livestock such as cattle, goats, sheep, horses, and mules (14, 15). This study was done in Benatsemay and South Ari woredas. The study was carried out from February 1 to 28, 2023.

### Population

All households that were found in Benatsemay and the South Ari districts served as the source population whereas randomly selected households in the sampled kebeles of Benatsemay and South Ari districts that meet the eligibility criteria were the study population. Households in the Benatsemay and South Ari districts, with heads who had resided in the study areas for a minimum of 6 months, were included in the study. However, households whose heads were unable to provide data due to serious illness or mental health issues were excluded.

### Sample size determination and sampling procedure

The sample size was determined using a single population proportion formula, assuming a confidence level of 95%, margin of error (d) of 5, 50% proportion of food insecurity (16), and 1.5 design effect. After considering a 5% non-response rate, the sample size used for this study was 605.

**Figure 1:**
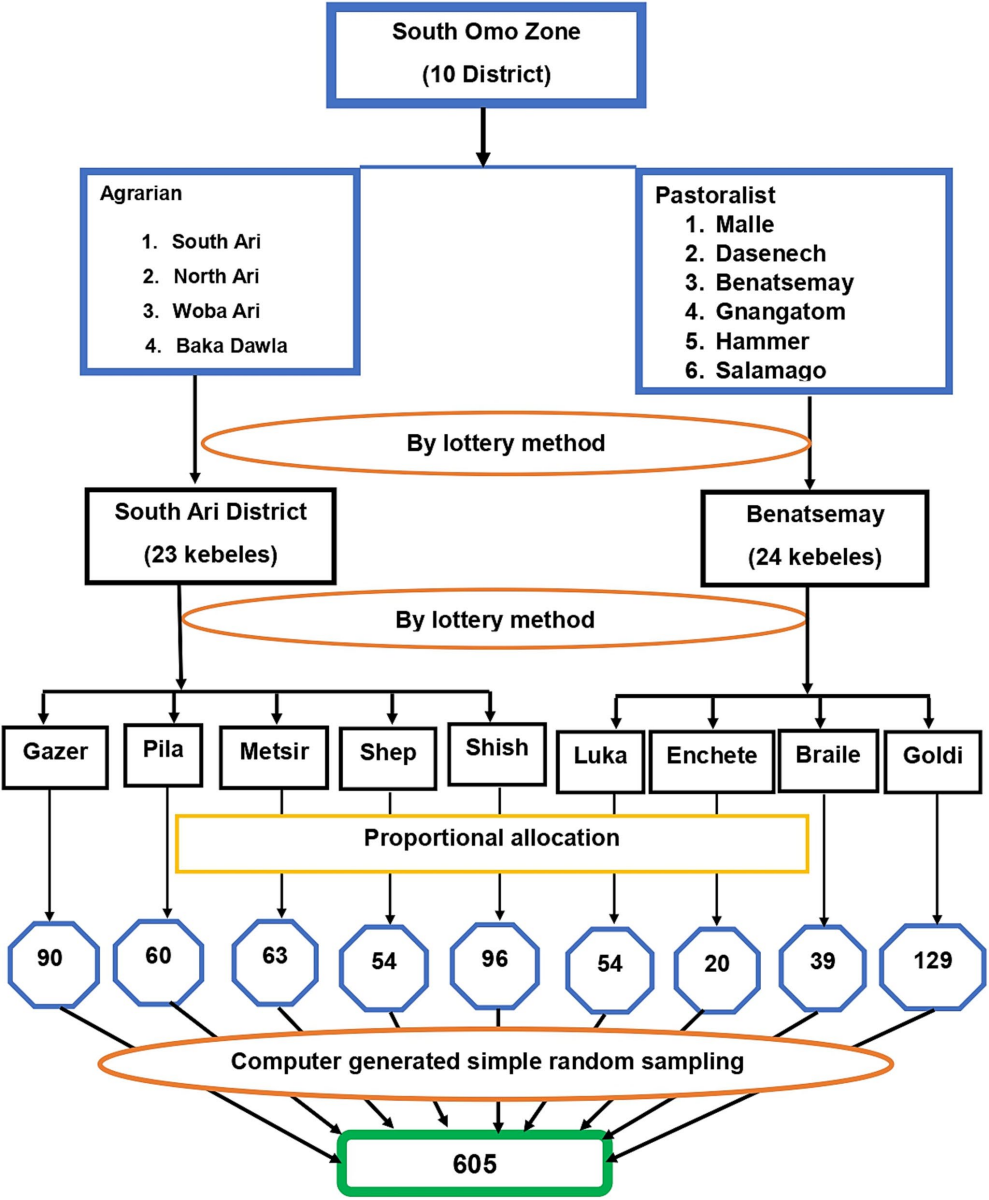
Schematic representation of the sampling procedure for the selection of study participants in South Omo Zone, Southern Ethiopia, 2023.

A multi-stage sampling method was employed to select the study subjects. Districts served as primary sample units, kebeles as secondary sample units, and households as tertiary sampling units. In Ethiopia, kebele is the district’s lowest administrative unit. First, 10 districts in the Zone were classified as pastoralist (*N* = 6) or agrarian (*N* = 4) based on their lifestyles. Lottery method was employed to select one district from each category, yielding the South Ari and Benatsemay districts. Then five kebeles from the South Ari Woreda and four kebeles from the Benatsemay district with a total of 9 kebeles were chosen by applying the lottery method. Household numbers for each kebele were determined using proportional allocation, based on the number of eligible households in each kebele. Study participants were chosen through computer-generated simple random sampling of households from the nine selected kebeles. A sampling frame of eligible households was created for each kebele by collecting information from the health post family folder and entering it into SPSS 25.0 software. Subjects were then randomly selected using the SPSS select case procedure (Figure 1).

### Study variables

Household food security level was considered as a dependent variable. Socio-demographic and economic factors such as maternal age, family size, sex of household head, age of household head, marital status, household wealth index, food insecurity, mother’s employment status, mother’s educational status, father’s education, dependency ratio, and residence; agriculture-related factors such as land ownership, farmland size and use of agricultural input; and service-related factors such as safety net program, access to credit, and agricultural extension were the independent variables.

### Data collection instrument, personnel, and procedure

Data were collected using a structured questionnaire, adapted from the Ethiopian Demographic and Health Survey (EDHS) and other relevant literature, aligning with the study’s objectives. First, the questionnaire was adapted into an English version and subsequently translated into Amharic. It included various types of information, including sociodemographic and economic details, household food insecurity, agricultural, and service-related factors. Nine nurses and one health extension worker from each kebeles were recruited to collect the data, and two masters of public health experts supervised the overall data collection process.

#### Food insecurity status

It was measured using a standardized and validated Household Food Insecurity Access Scale (HFIAS) instrument developed by FANTA version 3 (17). Nine questions about experiences of food in the household were posed to the mothers within the 30 days before the study. These were categorized into three primary areas of household food insecurity such as (1) insufficient food quality (3 questions), (2) inadequate food consumption and its physical effects (5 questions), and (3) anxiety and uncertainty regarding food access (1 question). The HFIAS tool contains nine “occurrence questions” (see Table 1), which reflect an increasing level of access conditions, and nine “frequency-of-occurrence” questions, which were responded to as a follow-up to each occurrence question to determine how frequently the condition occurred. After receiving yes or no answers to the questions, frequency questions were utilized to determine the four categories of food insecurity prevalence. Accordingly, these questions resulted in the food insecurity of households being classified as either food secure or food insecure. Households experienced none of the food insecurity (access) conditions, or just experienced worry, but rarely were categorized as food secure. Households worry about not having enough food sometimes or often, and/or households are unable to eat preferred foods, and/or households eat a more monotonous diet than desired, and/or some foods are considered undesirable, but only rarely but they did not experience three most severe conditions (running out of food, going to bed hungry, or going a whole day and night without eating) were considered as mildly food insecure. Households sacrifice quality more frequently, by eating a monotonous diet or undesirable foods sometimes or often, and/or have started to cut back on quantity by reducing the size of meals or the number of meals, rarely or sometimes. However, it does not experience any of the three most severe conditions that were considered moderately food insecure. Whereas, any household that experiences one of these three conditions even once in the last 4 weeks (30 days) is considered severely food insecure or has experienced cutting back on meal size or the number of meals often, and/or experiences any of the three most severe conditions (running out of food, going to bed hungry, or going a whole day and night without eating), even as infrequently as rarely were considered as severely food insecure (17). Mildly, moderately or severely food-insecure households were combined and considered as food insecure.

**Table 1:** Distribution of households based on the occurrence of food insecurity in pastoralist and agrarian communities of South Omo Zone, Southern Ethiopia, 2023 (*N* = 597).

Occurrence questions (*N* = 597)		Yes	No
		Frequency (%)	Frequency (%)
1	Worry about not having enough food to eat	227 (38.0)	370 (62.0)
1a	Rarely (1–2 times) Sometimes (3–10 times) Often (more than 10 times)	191 (84.1) 34 (15.0) 2 (0.9)	
2	Unable to eat preferred foods	208 (34.8)	389 (65.2)
2a	Rarely (1–2 times) Sometimes (3–10 times) Often (more than 10 times)	174 (83.7) 34 (16.3) 0 (0.0)	
3	Eating just a few kinds of foods	137 (22.9)	460 (77.1)
3a	Rarely (1–2 times) Sometimes (3–10 times) Often (more than 10 times)	107 (78.1) 30 (21.9) 0 (0.0)	
4	Eating some foods they really do not want to eat	79 (13.2)	518 (86.8)
4a	Rarely (1–2 times) Sometimes (3–10 times) Often (more than 10 times)	60 (75.9) 19 (24.1) 0	
5	Eating a smaller size of meals	90 (15.1)	507 (84.9)
5a	Rarely (1–2 times) Sometimes (3–10 times) Often (more than 10 times)	87 (96.7) 3 (3.3) 0 (0.0)	
6	Eating fewer meals in a day	113 (18.9)	484 (81.1)
6a	Rarely (1–2 times) Sometimes (3–10 times) Often (more than 10 times)	111 (98.2) 2 (1.8) 0 (0.0)	
7	No food to eat of any kind in the household	31 (5.2)	566 (94.8)
7a	Rarely (1–2 times) Sometimes (3–10 times) Often (more than 10 times)	22 (71.0) 9 (29.0) 0 (0.0)	
8	Go to bed hungry	31 (5.2)	566 (94.8)
8a	Rarely (1–2 times) Sometimes (3–10 times) Often (more than 10 times)	28 (90.3) 3 (9.7) 0 (0.0)	
9	Go a whole day and night without eating anything	14 (2.3)	583 (97.7)
9a	Rarely (1–2 times) Sometimes (3–10 times) Often (more than 10 times)	11 (78.6) 3 (21.4) 0 (0.0)	

#### Household wealth index

A combined indicator of the household’s overall standard of life. The easy-to-gather data on the ownership of a household’s 26 specific types of assets was utilized to generate the household wealth index (18). A statistical procedure called principal components analysis (PCA) was used to create the wealth index, which ranks individual households on a continuous scale of relative wealth. Each household asset was assigned a factor score derived from PCA. The succeeding asset scores were transformed into a normal distributed standard deviation with a mean of zero and a standard deviation of one. Then, standardized scores were used to generate the cutoff point that defines the household wealth index into tertile (poor, medium, and rich).

#### Dependency ratio

Measures the number of dependents in the household (those aged zero to 14 and those 65 and older) compared to the working-age population in the households (aged 15–64). It gives insight into the number of people of non-working age in the household, compared with the number of those of working age in the household. It is computed by dividing the number of dependents by the working-age population and then multiplying the result by 100. Then, the results were used to generate the cutoff point that defines the household dependency ratio into tertile (low, medium, and high) (19, 20).

### Data quality assurance

A structured questionnaire was initially created in English and subsequently translated into Amharic, the local language. It was then translated back to English by different translators to check for any inconsistencies. Data collectors and supervisors underwent a two-day training. After pretesting the questionnaire on 5% of the sample size in the Bakadawula and Malle districts, necessary adjustments were made. Daily supervision ensured that questionnaires were reviewed each day for completeness as well as consistency.

### Data processing and analysis

Data were entered and cleaned on Epi-Info version 7.1 and transferred to SPSS version 25 software for further analysis. Descriptive statistics were computed to describe all variables in the study. Food security was determined using HFIAS occurrence and frequency questions. Food security categories were constructed according to the following criteria set in the HFIAS guideline:

Food secure household: if [(Q1a = 0 or Q1a = 1) and Q2 = 0 and Q3 = 0 and Q4 = 0 and Q5 = 0 and Q6 = 0 and Q7 = 0 and Q8 = 0 and Q9 = 0], the household did not experience any of the food insecurity situations, or only had the experience of worrying about food but rather infrequently.

Mildly food insecure household: if [(Q1a = 2 or Q1a = 3 or Q2a = 1 or Q2a = 2 or Q2a = 3 or Q3a = 1 or Q4a = 1) and Q5 = 0 and Q6 = 0 and Q7 = 0 and Q8 = 0 and Q9 = 0], the household worries about not having food to eat occasionally or frequently, and/or being unable to consume choice foods, and/or having little variety of food, and/or some food referred to as unpalatable only on rare occasions.

Moderately food insecure household: if [(Q3a = 2 or Q3a = 3 or Q4a = 2 or Q4a = 3 or Q5a = 1 or Q5a = 2 or Q6a = 1 or Q6a = 2) and Q7 = 0 and Q8 = 0 and Q9 = 0], the household consumes few varieties or unpalatable foods occasionally or frequently, and/or has begun to reduce the size or number of meals infrequently or occasionally but did not experience any of the three extreme food insecurity situations.

Severely food insecure household: if [Q5a = 3 or Q6a = 3 or Q7a = 1 or Q7a = 2 or Q7a = 3 or Q8a = 1 or Q8a = 2 or Q8a = 3 or Q9a = 1 or Q9a = 2 or Q9a = 3], the household has moved gradually to reducing the quantity of meal or number of meals most frequently, and/or experiencing the three most extreme situations such as “not having any food to eat,” “going to bed without eating any food,” or “going a whole day hungry,” even infrequently.

Households that found mild, moderate, and severe forms of food insecurity were merged as food insecure. The dependent variable was coded with a “1” for food-insecure and a “0” for food-secure households. A binary logistic regression model was used to determine factors linked with food insecurity status. Results of bivariable analysis were presented using crude odds ratio (COR) with its conforming confidence interval (CI) of 95%. Independent variables that showed significant association at *p*-value <0.25 in bivariable analysis were entered into a model of multivariable logistic regression to determine factors. The enter method was used to fit a multivariable logistic regression model. The adjusted Odds Ratio (AOR) and its corresponding 95% confidence interval (CI) were used to assess the relationships between the independent and dependent variables. A *p*-value <0.05 was used to declare statistical significance in the final model.

Multicollinearity between independent variables was assessed for all candidate variables by using variance inflation factor (VIF) <10. The highest observed VIF-value in this study is <10, indicating no threat of multicollinearity. Hosmer–Lemeshow goodness-of-fit statistic was used to check model fitness and was satisfied (*p*-value ≥0.05).

## Results

### Socio-demographic and economic features of the respondents

A total of 597 respondents were successfully interviewed with a response rate of 98.7%. Over one-third of children’s mothers (36%) had no history of formal education and the majority (48.7%) were housewives. Also, above half (65.3%) were rural dwellings and the majority (33.7%) were under households with poor wealth index (Table 2).

**Table 2:** Socio-demographic and economic profiles of study participants in pastoralist and agrarian communities of South Omo Zone, Southern Ethiopia, 2023 (*N* = 597).

Variables	Categories	Frequency (*N*)	Percent (%)
Household head sex	Male	381	63.8
	Female	216	36.2
Household head age (in years)	20–24	30	5.0
	25–29	186	31.2
	30–34	162	27.1
	35–39	78	13.1
	40–44	78	13.1
	45–49	39	6.5
	50–54	18	3
	≥55	6	1
Household head educational status	No formal education	222	37.2
	Primary education	219	36.7
	Secondary education	75	12.6
	College/University	81	13.6
Maternal age (in years)	20–24	87	14.6
	25–29	249	41.7
	30–34	138	23.1
	35–39	75	12.6
	≥40	48	8
Marital status	Single	6	1.0
	Married	561	94.0
	Widowed	5	0.8
	Divorced	25	4.2
Religion	Orthodox	176	29.5
	Protestant	352	59.0
	Muslim	33	5.5
	Cultural	33	5.5
	Catholic	3	0.5
Ethnicity	Ari	253	42.4
	Amhara	79	13.2
	Woliata	49	8.2
	Goffa	12	2
	Bena	112	18.8
	Tsemay	92	15.4
Maternal education	No formal education	221	37
	Primary education	217	36.3
	Secondary education or higher	159	26.6
Maternal occupation	Daily laborer	45	7.5
	Employee	45	7.5
	Farmer	174	29.1
	Housewife	291	48.7
	Merchant	33	5.5
	Pastoralist	9	1.5
Fathers’ education	No formal education	185	31
	Primary education	220	36.9
	Secondary education	99	16.6
	College/university	93	15.6
Fathers’ occupation	Daily laborer	33	5.5
	Employee	90	15.1
	Farmer	276	46.2
	Pastoralist	54	9
	Merchant	138	23.1
	Others	6	1
Family size	02-Apr	288	48.2
	05-Jul	240	40.3
	≥8	69	11.5
Dependency ratio	Low	216	36.2
	Medium	219	36.7
	High	162	27.1
Household wealth index	Poor	201	33.7
	Medium	205	34.3
	Rich	191	32
Residence	Rural	390	65.3
			34.7
	Urban	207	

### Status of household food security and characteristics related to food insecurity

The HFIAS tool of nine occurrence questions for food insecurity among households in pastoralist and agrarian communities of South Omo Zone is found in Table 1. It showed that 62.0, 65.2, 77.1, and 86.8% of households responded “No” to the occurrence questions 1–4, whereas 84.9, 81.1, 94.8, 94.8, and 97.7% of household responded “NO” for the occurrence questions 5–9. The remaining households responded “Yes” to nine HFIAS occurrence questions. Table 1 indicates a decreasing trend in the households that responded favorably to the nine occurrence questions with a 4-week recall interval. Whereas, the number of households that responded “no” to the questions has steadily increased.

**Figure 2:**
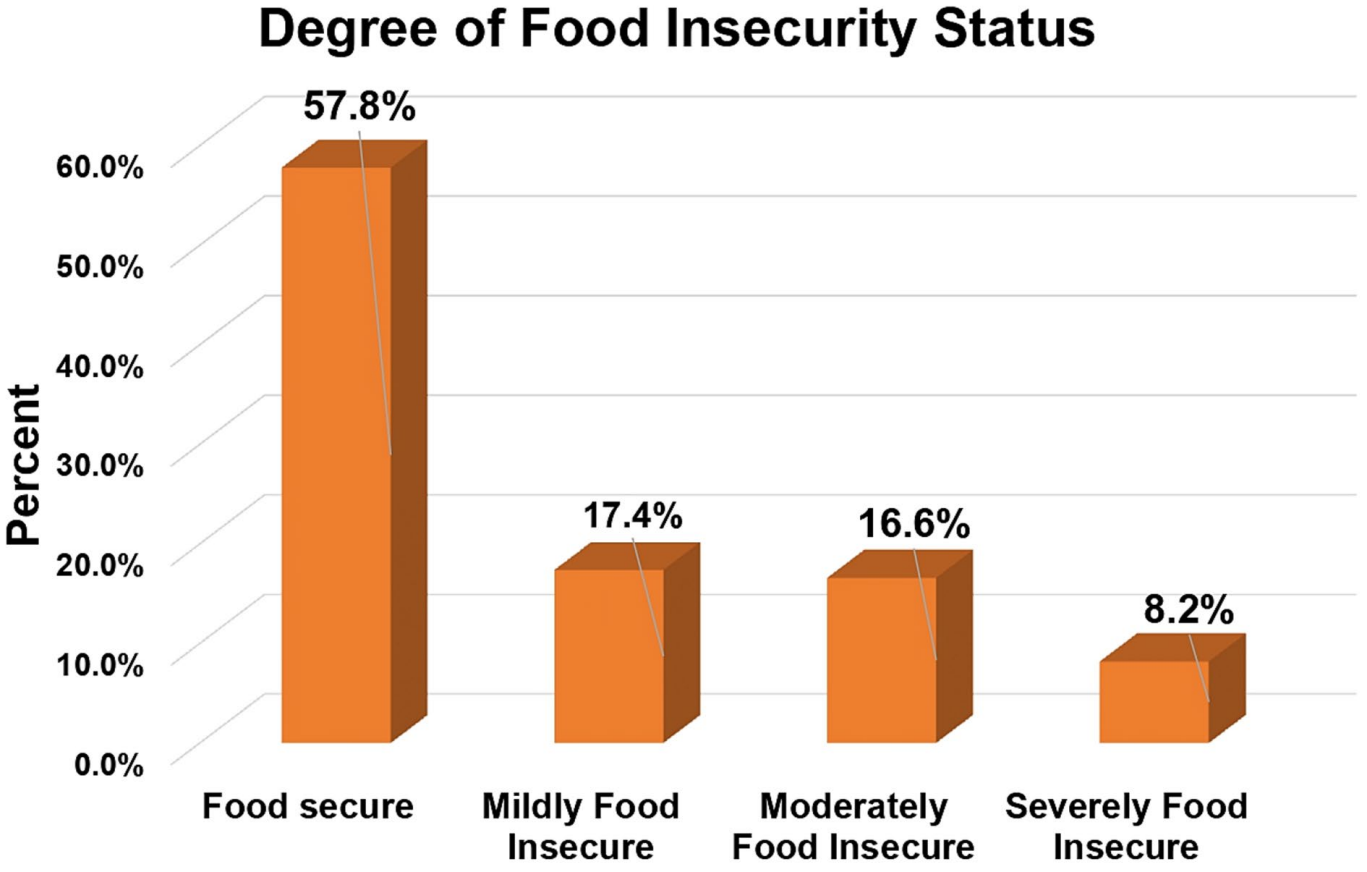
Level of household food insecurity status in South Omo Zone, Southern Ethiopia, 2023.

This study revealed that 42.2% (95% CI: 38.2, 46.3%) of the households found in pastoralist and agrarian areas of South Omo Zone were food insecure (Table 3). Among these mild, moderate, and severe food insecurity accounted for 104 (17.4%), 99 (16.6%), and 49 (8.2%), respectively (Figure 2).

**Table 3:** Status of household food security and characteristics related to food insecurity in the pastoralist and agrarian communities of South Omo Zone, Southern Ethiopia, 2023 (*N* = 597).

Variables		Frequency (*N*)	Percent (%)
Any family member migrated for work	Yes	63	10.6
	No	534	89.4
Land ownership	Yes	333	55.8
	No	264	44.2
Farmland Size (*n* = 333)	<1.5 hectare	218	65.5
	≥1.5 hectare	115	34.5
Use of agricultural input	Yes	160	26.8
	No	437	73.2
Use of agricultural extension service	Yes	94	15.7v
	No	503	84.3
Need food aid during the last year	Yes	57	9.5
	No	540	90.5
Use of productive safety-net service	Yes	45	7.5
	No	552	92.5
Household members change his/her food consumption over the past 12 months compared to the previous year	Yes	9	1.5
	No	588	98.5
Household experienced any shortage of food over the past 12 months	Yes	9	1.5
	No	588	98.5
Household food security status	Food secure	345	57.8
	Food insecure	252	42.2

**Figure 3:**
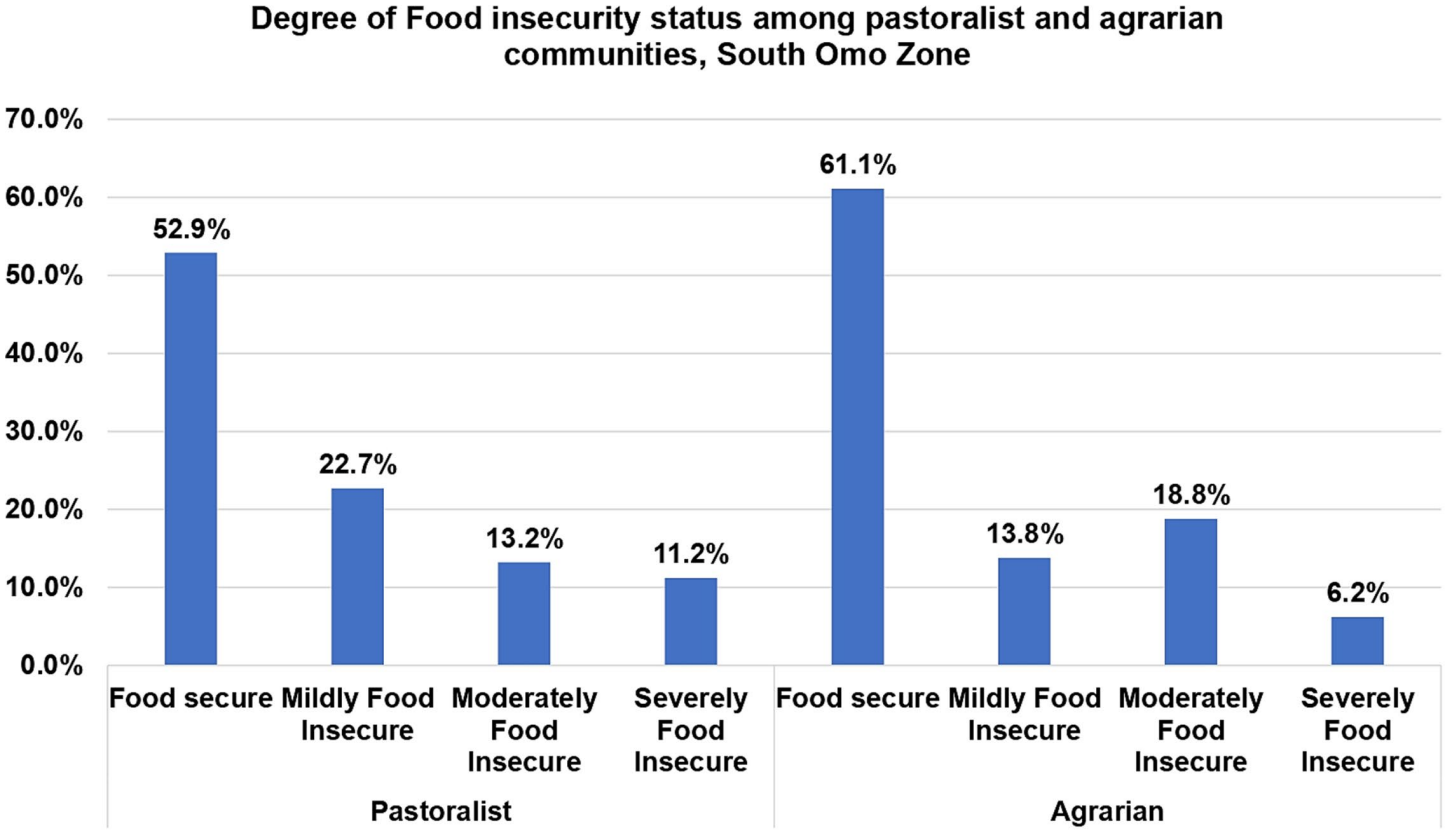
Degree of household food insecurity status among pastoralist and agrarian communities in South Omo Zone, Southern Ethiopia, 2023.

The prevalence of food insecurity among pastoralists was 114 (47.1%) whereas 138 (38.9%) were food insecure in the agrarian communities in South Omo Zone. Regarding the degree of food insecurity, 55 (22.7%), 32 (13.2%), and 27 (11.2%) were mildly, moderately, and severely food insecure, respectively, in the pastoralist communities. Whereas, 49 (13.8%), 67 (18.8%), and 22 (6.2%) were mildly, moderately, and severely food insecure, respectively, in the agrarian communities (Figure 3).

### Determinants of food insecurity

During the bivariable logistic regression model, household head sex, marital status, family size, maternal educational status, dependency ratio, household head education, household wealth index, productive safety-net program (PSNP) status, agricultural extension service use, land ownership, and use of agricultural input had significant associations with food insecurity at *p*-value <0.25. After controlling for confounding variables in the multivariable logistic regression model, household head sex, dependency ratio, maternal educational status, PSNP status, and land ownership were associated with the household’s food insecurity status at *p*-value<0.05.

Households with a female head were 1.73 times more food insecure as compared to households with a male head (AOR = 1.73, 95%CI: 1.14, 2.62). Households with a high dependency ratio were 2.53 times more likely to experience food insecurity compared to those with a low dependency ratio (AOR = 2.53, 95% CI: 1.53, 4.20). Similarly, medium dependency ratio households were 1.72 times more likely to face food insecurity than a lower dependency ratio household (AOR = 1.71, 95% CI: 1.12, 2.62). Households with mothers lacking formal education were more than twice as likely to experience food insecurity compared to those with mothers who had secondary education or higher (AOR = 2.11, 95% CI: 1.07, 4.18). Also, households with a mother who holds primary education were twice as food insecure as compared to households with mothers with secondary schooling and beyond (AOR = 2.00, 95%CI: 1.10, 3.60). PSNP status influenced household food insecurity; households without PSNP were twice as likely to be food insecure compared to those with PSNP (AOR = 2.00, 95% CI: 1.16, 3.46). Additionally, compared to other households, those without access to farming land had nearly twice as much food insecurity (AOR = 1.80, 95%CI: 1.19, 2.73) (Table 4).

**Table 4:** Bivariable and multivariable logistic regression models predicting the likelihood of household food insecurity in the pastoralist and agrarian communities of South Omo Zone, Southern Ethiopia, 2023 (*N* = 597).

Variables		Food security status		COR (95% CI)	*P*-value	AOR (95% CI)	*P*-value
		Food insecure	Food secure				
		*N* (%)	*N* (%)				
Sex of household head	Male	138 (36.2)	243 (63.7)	1	1	1	0.009*
	Female	114 (52.8)	102 (47.2)	1.97 (1.40, 2.76)	<0.001	1.73 (1.14, 2.61)	
Family size	02–4	117 (40.6)	171 (59.4)	1	0.16	1	0.7
	05–7	112 (46.7)	128 (53.3)	1.28 (0.91, 1.81)	0.27	1.09 (0.71, 1.67)	0.14
	≥8	23 (33.3)	46 (66.7)	0.73 (0.42, 1.27)		0.62 (0.33, 1.17)	
Dependency ratio	Low	71 (32.9)	145 (67.1)	1	0.049	1	0.013*
	Medium	92 (42.0)	127 (58.0)	1.48 (1.00, 2.19)	<0.001	1.71 (1.12, 2.62)	<0.001*
	High	89 (54.9)	73 (45.1)	2.49 (1.64, 3.79)		2.53 (1.53, 4.20)	
Maternal educational status	No formal education	106 (48.0)	115 (52.0)	2.01 (1.31, 3.08)	0.001	2.11 (1.07, 4.18)	0.031*
	Primary education	96 (44.2)	121 (55.8)	1.73 (1.13, 2.66)	0.012	2.00 (1.10, 3.60)	0.022*
	Secondary education or higher	50 (31.4)	109 (68.6)	1		1	
Educational level of household head	No formal education	108 (48.6)	114 (51.4)	2.20 (1.43, 3.38)	<0.001	1.55 (0.79, 3.04)	0.2
	Primary education	97 (44.3)	122 (55.7)	1.84 (1.20, 2.85)	0.006	1.25 (0.71, 2.20)	0.44
	Secondary education or higher	47 (30.1)	109 (69.9)	1		1	
Household wealth index	Poor	91(45.3)	110 (54.7)	1.72 (1.14,2.59)	0.01	1.06 (0.62, 1.81)	0.84
	Middle	99(48.5)	106 (51.5)	1.94 (1.29,2.92)	0.001	1.57 (0.99, 2.50)	0.06
	Rich	62 (32.5)	129 (67.5)	1		1	
PSNP status	No	228 (44.1)	289 (55.9)	1.84 (1.11, 3.06)	0.019	2.00 (1.16, 3.46)	0.013*
	Yes	24 (30.0)	56 (70.0)	1		1	
Agricultural extension service use	No	220 (43.7)	283 (56.3)	1.51 (0.95, 2.39)	0.082	1.23 (0.70, 2.16)	0.47
	Yes	32 (34.0)	62 (66.0)	1		1	
Land ownership	No	125 (47.3)	139 (52.7)	1.46 (1.05, 2.02)	0.024	1.80 (1.19, 2.73)	0.006*
	Yes	127 (38.1)	206 (61.7)	1		1	
Agricultural input use	No	199 (45.5)	238 (54.5)	1.69 (1.16, 2.47)	0.007	1.43 (0.89, 2.30)	0.14
	Yes	53 (33.1)	107 (66.9)	1			

*Significant at *p*-value < 0.05.

## Discussion

This study evaluated food insecurity and contributing factors in pastoralist and agrarian communities in the South Omo Zone of Ethiopia. The findings showed that 42.2% of households experienced food insecurity. Key factors linked to food insecurity, after adjusting for all other confounders, included the household head sex, maternal education level, dependency ratio, non-participation in productive safety net programs, and land ownership.

In the current study, 42.2% of households in pastoralist and agrarian communities of South Omo Zone had food insecurity. This finding aligns quite closely with a study carried out in Southern Ethiopia, where 44.8% of households were deemed food insecure (21). Moreover, the results are in line with studies on food insecurity in the Gojjam Zone in northern Ethiopia (43.25%) (1), eastern Ethiopia (41.7%) (22), and Wolaita Sodo (37.6%) (12). This study finding is higher than those from studies conducted in western Oromia (19.6%) (23), Dessie and Combolcha cities, north-central Ethiopia (33.1%) (24), Debre Berhan town, Central Ethiopia (32.4%) (25), and Arba Minch Town (30.2%) (26). Conversely, this study finding is notably lower than studies reported from Northeast Iran (56.79%) (27), Southeastern Iran (58.8%) (28), Maputo city, Mozambique (79%) (29), Sekela District (73.1%) (30), Areka Town (69.6%) (31). The discrepancies could emanate from variations in study settings, time factors, methodology, and differences in socioeconomic conditions or infrastructures. Besides, the majority of other studies focused on urban settings, while our study was conducted in pastoralist and agrarian contexts.

In this study, among food-insecure households, 8.2% faced severe food insecurity. This finding is lower than a survey done in South Ethiopia’s East Badawacho District, where 31.0% indicated severe food insecurity (32). This variance may be attributable to changes in ecological conditions across the study areas. Partly, this discrepancy in the findings might also be due to differences in seasonal variation, sample size, and study settings.

In this study, household head sex is a factor associated with food insecurity. Being in a household led by a female head increased the risk of food insecurity. This finding is reaffirmed by studies reported from Northeast Iran (27), Farta District (33), Sekela District (30), Eastern Ethiopia (22), and West Abaya District, Ethiopia (34). This could be explained because people in the study setting were agrarian and pastoralist and culturally the community primarily depends on cultivating farmland and cattle breeding, which is the main responsibility of males; however, households headed by females hardly cultivate and breed cattle. This could explain why female-headed households had higher levels of food insecurity.

In comparison to households with a low reliance ratio, those with a high or moderate dependency ratio were found to be more food insecure in the current study. This finding aligns with a study from South Ari District, Ethiopia, which found that households with a high dependency ratio were more than twice as likely to face food insecurity than those with a low dependency ratio (21). Similarly, another study done in southwest Ethiopia revealed that households having a high dependency ratio faced a greater proportion of food insecurity compared to their counterparts (35). This is also supported by studies conducted in Southeastern Nigeria (36) and Ethiopia (20). It is evident that when the dependency ratio rises, the family’s working members have an increased responsibility to provide food, which raises the possibility of food insecurity.

Households with mothers without formal education were twice as food insecure than households with mothers with secondary education and above. This finding is reaffirmed by a study in Rwanda, which showed that households with a mother with no formal education had a 4.58 times more chance to experience food insecurity compared to those with mothers who had tertiary education (37). Similarly, research in Brazil also supports this trend, demonstrating that households with mothers with better education face a reduced chance of food insecurity when compared to less educated mothers (38). These findings underscore maternal education’s importance in mitigating household food insecurity, as educated mothers possess greater social and human capital to address these issues.

The presence of PSNP has determined the food insecurity of households; those households without Safety Net Program had a doubled likelihood of food insecurity when compared to those who had the Safety Net Program. Similarly, a study conducted on food insecurity and responsible determinants in South Ari Woreda reported that the odds of food insecurity were two-fold greater among non-users of PSNP than their counterparts (21). Similarly, research on the effect of PSNP on food security in western Ethiopia supports this conclusion, revealing that households not enrolled in the program faced a higher chance of food insecurity compared to those receiving Safety Net Program assistance (39). It is evident that the PSNP, whether through job creation or cash assistance, plays a crucial role in enhancing household food security.

Furthermore, households lacking farming land were twice as food insecure than households with land ownership. This finding is substantiated by a study done in the Oromia zone, Ethiopia, which showed that households owning their land faced reduced odds of food insecurity compared to those without land ownership (40). Similarly, research on food insecurity linked to household characteristics and agricultural practices in Madagascar indicated that households with smaller land holdings are at greater odds of food insecurity than those with larger plots (41). Households that own land have the advantage of retaining all harvests for their consumption without the need to share them with external entities.

### Limitations of the study and areas for further research

Despite the strengths, this study has some limitations. Because the study utilized a cross-sectional design, it was impossible to determine relevant temporal relationships. Although probing techniques and associations with known events were used to reduce recall bias during data collection, there may still be some degree of recall bias for past events, such as when asking about food security status from 4 weeks prior. The study was done only in two districts in the South Omo Zone, which might limit the generalizability of the findings to other pastoralist and agrarian areas. Moreover, the study did not consider the effect of seasonal variation on household food insecurity.

## Conclusion

This study attested that nearly three in every seven households experienced food insecurity in pastoralist and agrarian communities of South Omo Zone, Southern Ethiopia. Determinants such as being a female household head, high to medium dependency ratio, low maternal education, non-participation in productive safety-net programs, and lack of land ownership were identified as significantly linked with food insecurity. Hence, tailored interventions are required from government bodies, policymakers, and other stakeholders to address the burden of food insecurity. Promoting women’s education, advocating for family planning, upgrading agricultural technology for those with limited land holdings, and increasing the accessibility of safety net programs are highly demanding. Further study using a strong design is required to see the temporal relationship by considering the effect of seasonal variation on household food insecurity. Moreover, further qualitative research is needed to explore factors contributing to household food insecurity.

## Data Availability

The raw data supporting the conclusions of this article will be made available by the authors, without undue reservation.
